# Supplementary material for: BayesKAT: bayesian optimal kernel-based test for genetic association studies reveals joint genetic effects in complex diseases
Source: Brief Bioinform. 2024 Apr 22;25(3):bbae182. doi: 10.1093/bib/bbae182 (PMC11036342; doi:10.1093/bib/bbae182)
Supplement: BIB_Supplementary_Files_Final_bbae182 [file bib_supplementary_files_final_bbae182.pdf]

Supplementary Files

BayesKAT: Bayesian Optimal Kernel-based Test for genetic association studies reveals joint genetic effects in complex diseases

Sikta Das Adhikari [1,2], Yuehua Cui [1*], Jianrong Wang [2*] ,

[1]Department of Statistics and Probability, Michigan State University, East Lansing, MI 48824, USA.

[2]Department of Computational Mathematics, Science and Engineering, Michigan State University, East Lansing, MI 48824, USA.

Contents

0.1 Kernel functions 2
0.2 Importance of choosing appropriate Kernel 3
0.3 Comaparison between BayesKAT-MCMC and BayesKAT-MAP 3
0.4 Multiple testing correction 4
0.5 ADNI Data Handling and Pre-processing steps 5
0.6 Further Discussion 5

List of Tables

S1 Empirical power of BayesKAT-MCMC and BayesKAT-MAP 4
S2 List of significant genes selected by BayesKAT and their posterior probability of association 7
S3 List of the significant KEGG pathways selected by BayesKAT and their corresponding posterior probabilities of association. 8

List of Figures

S1 Performance comparison based on simulations involving unrelated discrete genetic features. With the same fixed level of empirical type 1 error, the empirical power of all three methods are overall low, because this basic simulation only groups unrelated genetic features together, which is not recommended for real-world genetic association studies. Even through, BayesKAT still achieves better empirical power. 9

S2 Performance comparison based on simulations involving unrelated discrete genetic features. With the same fixed level of empirical type 1 error, the empirical power of all three methods are overall low, because this basic simulation only groups unrelated genetic features together, which is not recommended for real-world genetic association studies. Even through, BayesKAT still achieves better empirical power. 10

S3 The empirical power vs empirical type 1 error plot from the pathway-based simulations with sample size n=755. For each scenario, BayesKAT consistently achieves the best performance. The aggregated result presented in main figure 3(B) 11

S4 The empirical power vs empirical type 1 error plot from the pathway-based simulations with sample size n=1000. The strength of the relationship is intentionally weakened by adjusting effect sizes for performance comparison purposes. Note that for larger sample sizes under the scenarios in S3, all methods exhibit a power of 1, making them incomparable. For each scenario, BayesKAT consistently achieves the best performance. 12

S5 The empirical power vs empirical type 1 error plot from the pathway-based simulations with sample size n=1500. The strength of the relationship is intentionally weakened by adjusting effect sizes for performance comparison purposes. Note that for larger sample sizes under the scenarios in S3, all methods exhibit a power of 1, making them incomparable. BayesKAT consistently performs reasonably well for each scenario. 13

0.1 Kernel functions

A kernel function is defined as a function $K : \mathcal{X} \times \mathcal{X} \to \mathbb{R}$, where the kernel matrix $K = (k_{i,i'})_{i,i'=1}^{n}$ is symmetric and positive semidefinite with $k_{i,i'} = k(Z_i, Z_{i'})$. In this setting, $k(Z_i, Z_{i'})$ is a measure of similarity between the ith and the i'th subject. There are a variety of kernel functions to choose from, and the most widely used ones include the Linear kernel, the Quadratic kernel and the Gaussian kernel. For genetic SNP data, identity by state (IBS) kernel is a popular candidate kernel function suggested by various studies [1], [2], [3]. The functional forms of these kernels are summarized below:

- Linear kernel: $K(Z_i, Z_{i'}) = Z_i^T Z_{i'}$
- Quadratic kernel: $K(Z_i, Z_{i'}) = (Z_i^T Z_{i'} + 1)^2$
- Gaussian kernel: $K(Z_i, Z_{i'}) = exp\{-\|Z_i - Z_{i'}\|^2/l\}$, where $\|Z_i - Z_{i'}\|^2 = \sum_{j=1}^{p}(Z_{ij} - Z_{i'j})^2$, l is a tuning parameter.
- IBS kernel: $K(Z_i, Z_{i'}) = (2p)^{-1}\sum_{j=1}^{p} IBS(Z_{ij}, Z_{i'j}) = (2p)^{-1}\sum_{j=1}^{p}(2 - |Z_{ij} - Z_{i'j}|)$

0.2 Importance of choosing appropriate Kernel

Although a variety of different kernels are available, for a given dataset, it is practically impossible to know a priori which kernel will fit the dataset best and maximize the testing power. Genetic data related to complex phenotypes pose particular challenges, primarily stemming from our limited understanding of how the interplay among genetic or molecular features influences their collective association with a phenotype. Therefore, choosing a kernel randomly can lead to a less powerful testing procedure for genome-wide applications. For example, if the outcome variable Y is related to the features through a Quadratic function, using a Linear kernel in the model will lead to weak tests that are not able to reject the null hypothesis even when the association is strong. On the other hand, by repeatedly applying KBT models based on different candidate kernels and choosing the one resulting in the minimum p-value, there is a high chance of making a false discovery, i.e., rejecting the null hypothesis when there is no association.

Combining a panel of candidate kernels together to create a composite kernel is thus a natural and effective strategy to overcome this issue. While a straightforward strategy of averaging kernels to form a composite kernel, i.e., a linear combination of candidate kernels with equal weights, can perform better than the worst-performing kernel function, it usually cannot perform as efficiently as the best kernel to accurately represent the association between the trait and features for a given dataset, thus, is not guaranteed to increase the statistical power. As shown in main Figure 1 (A)(B), evaluated on both real and synthetic datasets, the average kernel strategy can often lead to incorrect and inconsistent results in practice. Thus, a systematic data-adaptive approach of optimal kernel selection is highly desirable for high-dimensional genome-wide association tests, especially for complex human disease phenotypes that are genetically modulated by multiple inter-dependent genetic variants.

0.3 Comaparison between BayesKAT-MCMC and BayesKAT-MAP

The performance and runtime of BayesKAT-MCMC and BayesKAT-MAP under different settings are systematically compared. Supplementary Table S1 summarizes the

empirical type-1 error and empirical power for different simulated functional dependencies. The simulations were based on the proposed settings used by previous studies [4]. As shown in Table 1, both MCMC and MAP strategies achieve nearly equal statistical power in detecting associations. While BayesKAT-MCMC provides more information on the posterior distributions of parameters, it is more computationally expensive compared to BayesKAT-MAP. When the number of samples or features, i.e. n or p, increases beyond 500, BayesKAT-MCMC is not sufficiently scalable without requesting more high-performance computing resources. On the other hand, Main Figure 1(D) shows the superior computational scalability of BayesKAT-MAP based on the same level of computational resource support. Therefore, these two alternative strategies provide similar accuracy and complementary signatures for genetic association tests of complex traits, with BayesKAT-MAP being more flexible and conservative. For the rest of the paper, results of BayesKAT-MAP are presented due to its scalability. The code for implementing BayesKAT-MCMC and BayesKAT-MAP are both made publicly available via GitHub: https://github.com/wangjr03/BayesKAT.

Table S1: Empirical power of BayesKAT-MCMC and BayesKAT-MAP

h(Z)	n	p	BayesKAT-MCMC	BayesKAT-MAP
$h(Z) = 0$	500	500	0.018	0
$h(Z) = 2 \times Z_1 Z_3$	500	500	0.946	0.946
$h(Z) = 2 \times Z_1 Z_3 + 0.04 \times Z_i + 0.04 \times Z_3$	500	500	0.948	0.958
$h(Z) = 0.4 \times (Z_1 - Z_3) + 0.4 \times cos(Z_3) exp(-Z_3^2/5)$	500	500	0.976	0.998

The functional forms in simulation scenarios are taken from [4] and coefficients are adjusted based on n (sample size) and p (no. of features)

0.4 Multiple testing correction

When m_1 multiple groups are simultaneously tested, multiplicity corrections are needed. Multiple testing corrections on p-values from frequentist models are carried out using methods such as the Bonferroni correction [5], which controls the family-wise type 1 error to α by setting the individual test's type 1 error at α/m_1. Other methods like FDR control have also been popular ones. For multiple testing corrections on Bayesian models, the multiplicity control is achieved by setting a high prior probability of the individual null hypothesis, as suggested by previous studies [6]. For each test, the prior probability is set as $P(H_0) = 0.99$, which is equivalent to assuming that, on average, one in 100 tests is believed to have a true association. Considering the number of SNP groups in genetic applications, such as the number of biological pathways or co-expression modules, this choice of prior probability setting is regarded as rather conservative and ensures fair performance comparisons.

0.5 ADNI Data Handling and Pre-processing steps

The Alzheimer's Disease Neuroimaging Initiative(ADNI) data referenced in this manuscript is not publicly accessible. Interested users can request access through the official portal at https://adni .loni.usc.edu/data-samples/access-data/. Once granted access, users can navigate to the "downloads" section and download the genotype data. For this study, "ADNI 1 SNP genotype data - PLINK" file containing the .bed, .fam and .bim files was downloaded. Initially, these files contain information on 757 individuals and 620901 SNPs. The standard quality control steps are performed using Plink[7] software, which resulted in the removal of SNPs and individuals failing standard missingness thresholds, Minor Allele Frequency (MAF), and Hardy-Weinberg Equilibrium (HWE) criteria. Subsequent to quality control, a simple imputation technique addresses the remaining missing values, resulting in a final genotype matrix featuring 531086 SNPs and 756 individuals meeting the established quality criteria. Demographic and phenotypic information for these individuals is retrieved using the publicly available ADNIMERGE package (detailed description: https://adni.bitbucket.io/). Covariates, specifically Age, gender, and education levels, which exhibit significant linear relationships with the phenotype of interest (whole brain volume), are integrated into the model. By aligning individual IDs across response, genotype, and covariate data, a final dataset comprising 755 individual-level data points is obtained. The GitHub repository https://github.com/wangjr03/BayesKAT contains the specific codes for preprocessing real individual-level genotype datasets.

0.6 Further Discussion

In recent years, different deep learning models have been developed and applied in genomics studies to predict molecular features, such as gene expression, histone marks, chromatin accessibility and transcription factor binding, using DNA sequences as features [8], [9], [10], [11]. These models allow for in silico mutations of DNA sequences and the prediction of perturbed molecular features for each mutation individually. Compared to these models, the power of BayesKAT in delineating the collective group-level genetic associations based on automatic composite kernel selection opens up a new level of analytical capability of dissecting the genetic complexity. Combined together, the complementary advantages of BayesKAT and deep learning models are expected to facilitate novel mechanistic insights into human diseases.

The implementation of BayesKAT is not limited to genetic studies based on features of SNPs or gene expressions. Any group of continuous or discrete features that are functionally related can be tested for associations with an outcome using the BayesKAT methodology. For instance, BayeskAT can be used to test if a group of images is associated with a particular disease trait by adopting properly defined kernels. As another

direction for future developments, non-linear functions of candidate kernels, instead of the linear combinations, will be explored as the composite kernel, which may lead to improved power for kernel-based testing.

Table S2: List of significant genes selected by BayesKAT and their posterior probability of association

Gene Name	p(H1|Data)
CARD10	0.9835514
MGAT5	0.9474112
TMEM71	0.9237384
FAM174B	0.9042780
ACTA2	0.8677653
C18orf45	0.8569289
TMEM163	0.8357041
LRP1B	0.8257515
SLC35B4	0.8248786
EDNRA	0.7878737
ABCB7	0.7699132
C9orf135	0.7660853
SLC25A18	0.7583290
DKK2	0.7501011
PCID2	0.7387756
TMEM38A	0.7042073
NXNL2	0.7037605

Table S3: List of the significant KEGG pathways selected by BayesKAT and their corresponding posterior probabilities of association.

Pathway Name	p(H1|Data)
Pathways of neurodegeneration - multiple diseases	0.9998840
Alzheimer disease	0.9982576
Salmonella infection	0.9897521
Antifolate resistance	0.9666899
Huntington disease	0.9630876
Bile secretion	0.9530779
Alcoholic liver disease	0.9285561
Dilated cardiomyopathy	0.9240475
Metabolic pathways	0.9211165
Amyotrophic lateral sclerosis	0.9070785
Hypertrophic cardiomyopathy	0.8975824
Cardiac muscle contraction	0.8966726
mTOR signaling pathway	0.8869947
cAMP signaling pathway	0.8688107
Pathogenic Escherichia coli infection	0.8645842
Calcium signaling pathway	0.7962372
ABC transporters	0.7851350
Citrate cycle (TCA cycle)	0.7766141
Non-alcoholic fatty liver disease	0.7676582
AMPK signaling pathway	0.7640913
Parkinson disease	0.7242965

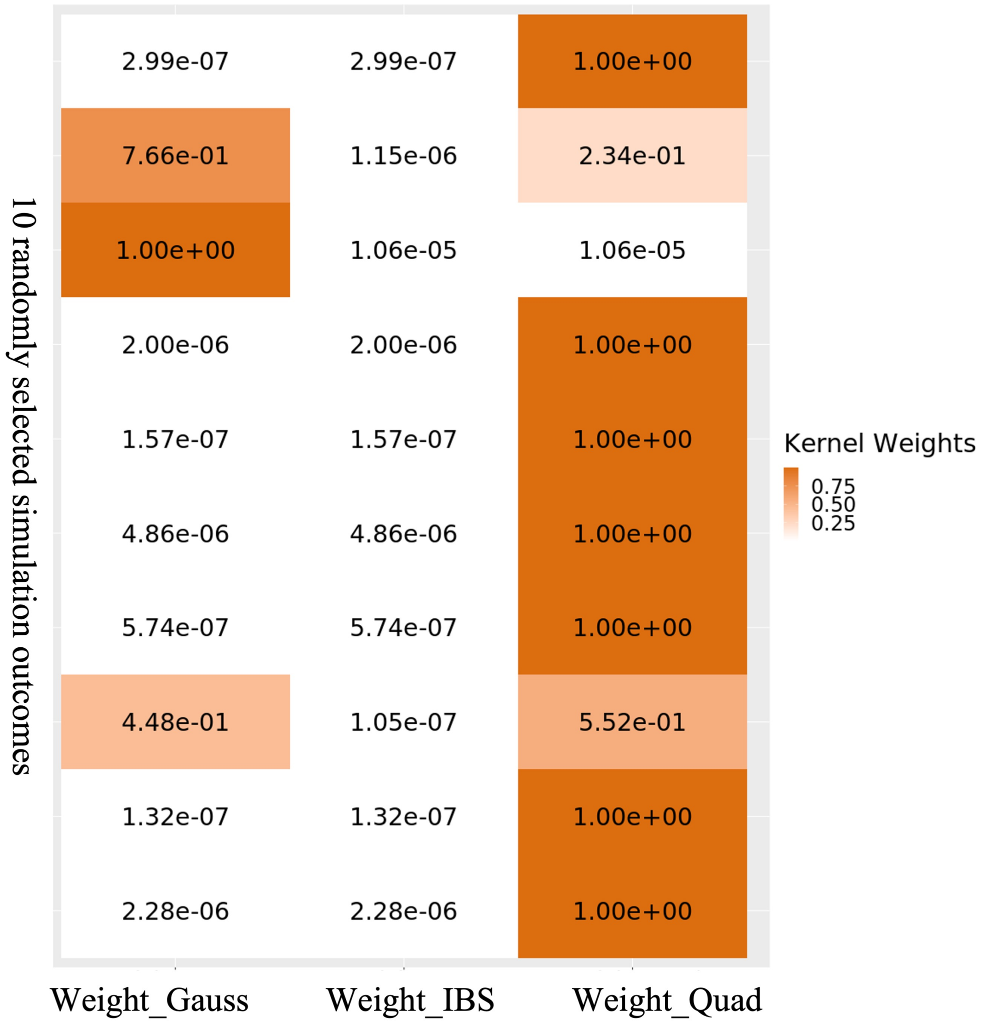

Figure S1: Performance comparison based on simulations involving unrelated discrete genetic features. With the same fixed level of empirical type 1 error, the empirical power of all three methods are overall low, because this basic simulation only groups unrelated genetic features together, which is not recommended for real-world genetic association studies. Even through, BayesKAT still achieves better empirical power.

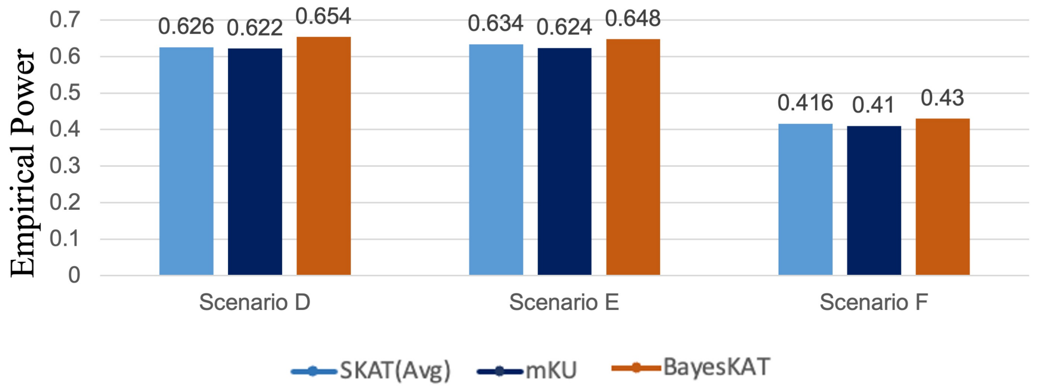

Figure S2: Performance comparison based on simulations involving unrelated discrete genetic features. With the same fixed level of empirical type 1 error, the empirical power of all three methods are overall low, because this basic simulation only groups unrelated genetic features together, which is not recommended for real-world genetic association studies. Even through, BayesKAT still achieves better empirical power.

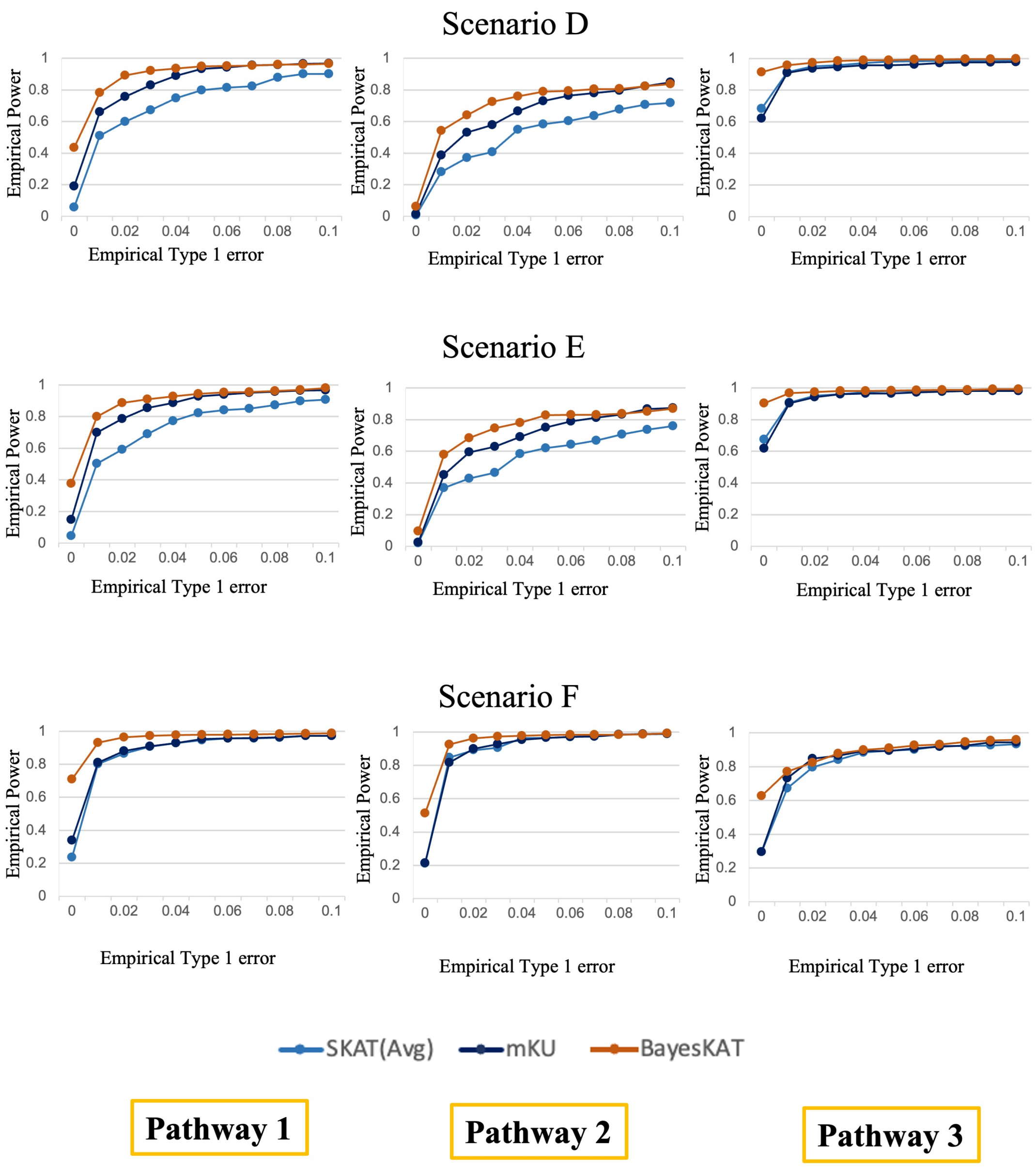

Figure S3: The empirical power vs empirical type 1 error plot from the pathway-based simulations with sample size n=755. For each scenario, BayesKAT consistently achieves the best performance. The aggregated result presented in main figure 3(B)

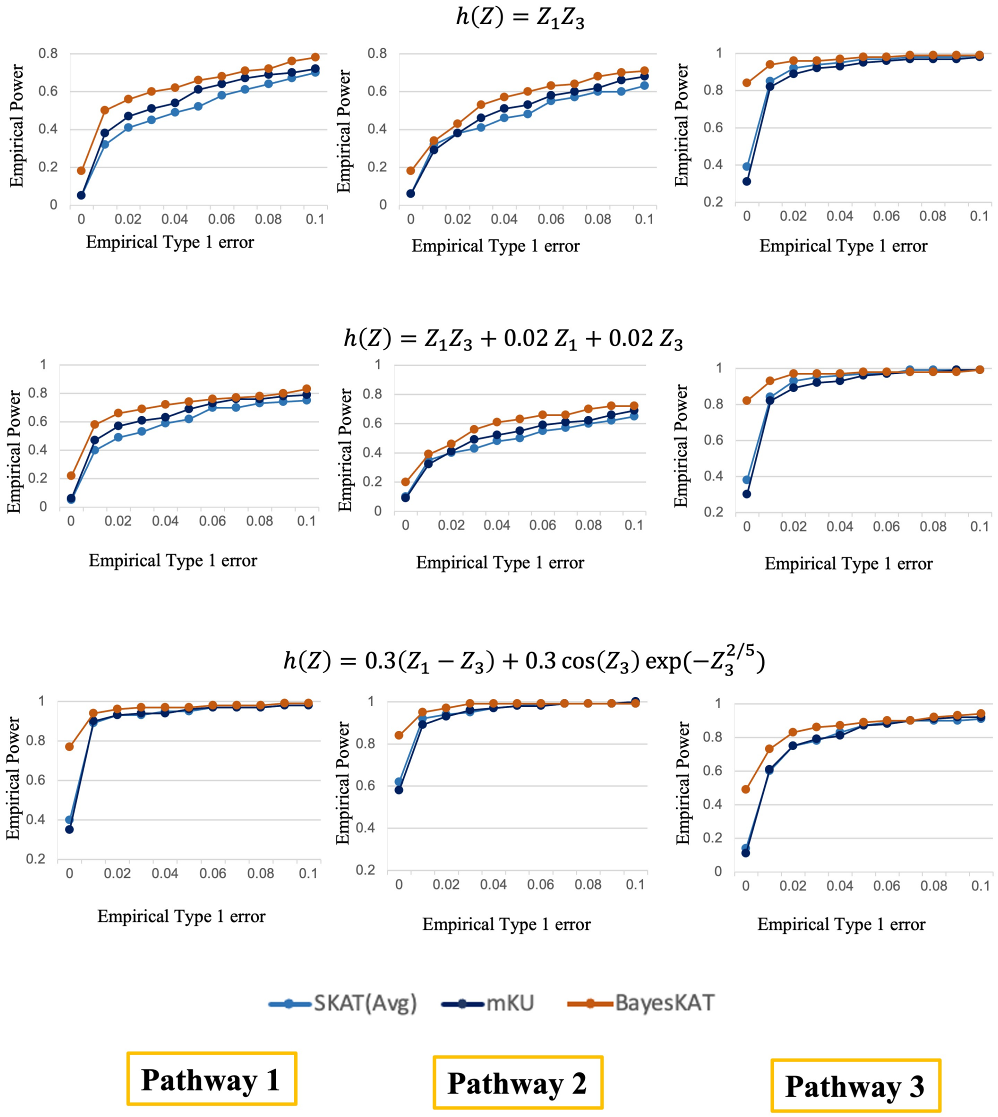

Figure S4: The empirical power vs empirical type 1 error plot from the pathway-based simulations with sample size n=1000. The strength of the relationship is intentionally weakened by adjusting effect sizes for performance comparison purposes. Note that for larger sample sizes under the scenarios in S3, all methods exhibit a power of 1, making them incomparable. For each scenario, BayesKAT consistently achieves the best performance.

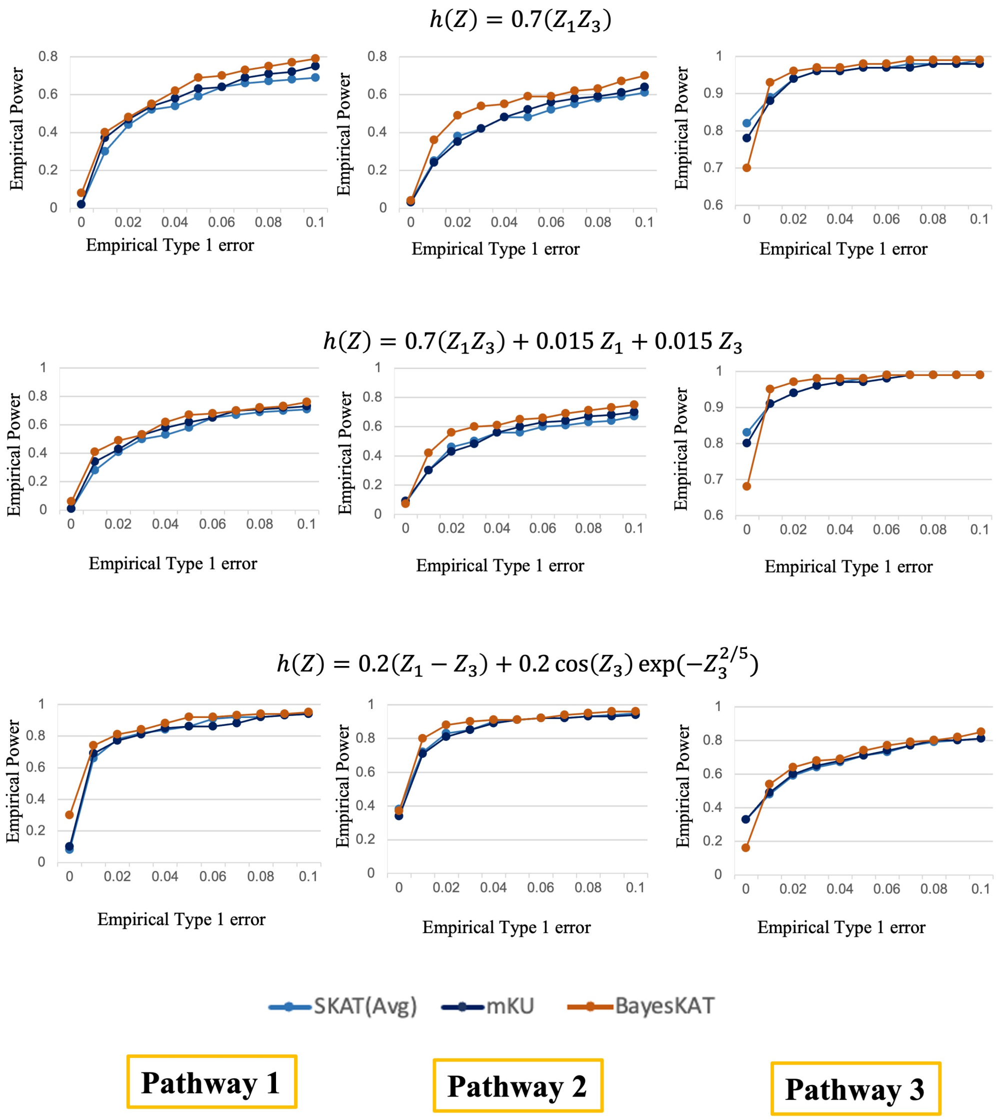

Figure S5: The empirical power vs empirical type 1 error plot from the pathway-based simulations with sample size n=1500. The strength of the relationship is intentionally weakened by adjusting effect sizes for performance comparison purposes. Note that for larger sample sizes under the scenarios in S3, all methods exhibit a power of 1, making them incomparable. BayesKAT consistently performs reasonably well for each scenario.

References

[1] Jennifer Wessel and Nicholas J Schork. Generalized genomic distance–based regression methodology for multilocus association analysis. *The American Journal of*

Human Genetics, 79(5):792–806, 2006.

[2] Lydia Coulter Kwee, Dawei Liu, Xihong Lin, Debashis Ghosh, and Michael P Epstein. A powerful and flexible multilocus association test for quantitative traits. *The American Journal of Human Genetics*, 82(2):386–397, 2008.

[3] Michael C. Wu, Seunggeun Lee, Tianxi Cai, Yun Li, Michael Boehnke, and Xihong Lin. Rare-variant association testing for sequencing data with the sequence kernel association test. *The American Journal of Human Genetics*, 89(1):82–93, 2011.

[4] Tao He, Shaoyu Li, Ping-Shou Zhong, and Yuehua Cui. An optimal kernel-based u-statistic method for quantitative gene-set association analysis. *Genetic epidemiology*, 43(2):137–149, 2019.

[5] Bradley Efron. *Large-scale inference: empirical Bayes methods for estimation, testing, and prediction*, volume 1. Cambridge University Press, 2012.

[6] Jon Wakefield. Bayes factors for genome-wide association studies: comparison with p-values. *Genetic Epidemiology*, 33(1):79–86, 2009.

[7] Shaun Purcell, Benjamin Neale, Kathe Todd-Brown, Lori Thomas, Manuel A R Ferreira, David Bender, Julian Maller, Pamela Sklar, Paul I W de Bakker, Mark J Daly, and Pak C Sham. PLINK: a tool set for whole-genome association and population-based linkage analyses. *Am J Hum Genet*, 81(3):559–575, July 2007.

[8] Jian Zhou and Olga G Troyanskaya. Predicting effects of noncoding variants with deep learning–based sequence model. *Nature methods*, 12(10):931–934, 2015.

[9] Jian Zhou, Chandra L Theesfeld, Kevin Yao, Kathleen M Chen, Aaron K Wong, and Olga G Troyanskaya. Deep learning sequence-based ab initio prediction of variant effects on expression and disease risk. *Nature genetics*, 50(8):1171–1179, 2018.

[10] David R Kelley, Jasper Snoek, and John L Rinn. Basset: learning the regulatory code of the accessible genome with deep convolutional neural networks. *Genome research*, 26(7):990–999, 2016.

[11] Žiga Avsec, Vikram Agarwal, Daniel Visentin, Joseph R Ledsam, Agnieszka Grabska-Barwinska, Kyle R Taylor, Yannis Assael, John Jumper, Pushmeet Kohli, and David R Kelley. Effective gene expression prediction from sequence by integrating long-range interactions. *Nature methods*, 18(10):1196–1203, 2021.
